# The Beneficial Effects of Combined Grape Pomace and Omija Fruit Extracts on Hyperglycemia, Adiposity and Hepatic Steatosis in *db*/*db* Mice: A Comparison with Major Index Compounds

**DOI:** 10.3390/ijms151017778

**Published:** 2014-09-30

**Authors:** Su-Jung Cho, Hae-Jin Park, Un Ju Jung, Hye-Jin Kim, Byoung Seok Moon, Myung-Sook Choi

**Affiliations:** 1 Department of Food Science and Nutrition, Kyungpook National University, 1370 Sankyuk Dong Puk-ku, Daegu 702-701, Korea; E-Mails: chosj1181@naver.com (S.-J.C.); purin613@hotmail.com (H.-J.P.); 2 Center for Food and Nutritional Genomics Research, Kyungpook National University, 1370 Sankyuk Dong Puk-ku, Daegu 702-701, Korea; E-Mail: jungunju@naver.com; 3 Food Research & Development, CJ Cheiljedang Corporation, Seoul 152-051, Korea; E-Mails: hyejink@cj.net (H.-J.K.); moonbs@cj.net (B.S.M.)

**Keywords:** grape pomace, omija fruit, hyperglycemia, adiposity, hepatic steatosis

## Abstract

This study investigated the effects of combined grape pomace and omija fruit extracts (GO) on diabetes-related metabolic changes in type 2 diabetic *db*/*db* mice. The effects of GO were compared with those of a resveratrol and schizandrin mixture (RS), which is a mixture of major components of GO. Mice were fed a normal diet with RS (0.005% resveratrol and 0.02% schizandrin in diet, *w*/*w*) or GO (0.3% grape pomace ethanol extract and 0.05% omija fruit ethanol extract in diet, *w*/*w*) for seven weeks. RS and GO not only lowered the levels of blood and plasma glucose, HbA_1c_, insulin and homeostasis model assessment of insulin resistance (HOMA-IR) with a simultaneous decrease in hepatic gluconeogenic enzymes activities and adiposity, but also improved preservation of the pancreatic β-cells. Plasma leptin and resistin levels were lower while the plasma adiponectin level was higher in the RS and GO groups than in the control group. Especially, GO increased hepatic glucokinase activity and gene expression and improved hepatic steatosis by elevating fatty acid oxidation compared to RS. These findings suggest that GO ameliorates hyperglycemia, adiposity and hepatic steatosis in type 2 diabetic mice.

## 1. Introduction

Type 2 diabetes is one of the most common metabolic disorders. The pathogenesis of type 2 diabetes is characterized by a combination of impaired insulin secretion from pancreatic β-cells and insulin resistance. In particular, hepatic insulin resistance has been suggested as a main factor in the development of hyperglycemia, because the liver centrally controls glucose production and utilization [1]. Hepatic insulin resistance is associated with hepatic steatosis, which is highly prevalent in patients with type 2 diabetes [2,3].

Grapes contain numerous polyphenols, including resveratrol, quercetin, catechins and anthocyanins, which have been shown to reduce blood glucose and improve pancreatic β-cell function [4]. In grapes, grape pomace was shown to reduce fasting plasma glucose, insulin and triglyceride levels in growing Sprague-Dawley rats [5]. As another blood glucose controller, omija (*Schisandra chinensis* Baillon), a traditional Chinese alternative medicine, has been used as an anti-diabetic agent in Asia [6].

In our previous study, we showed that combined grape pomace and omija fruit extracts (GO) ameliorated adipogenesis and hepatic steatosis in high-fat diet (HFD)-induced obese mice [7]. We have investigated here if GO would not only lower body fat mass, but also improve diabetes-related metabolic changes, including plasma biomarkers, β-cell failure and hepatic glucose-regulating enzymes in *db*/*db* mice. These mice display many of the characteristics of type 2 diabetes, including hyperphagia, hyperglycemia, β-cell loss and insulin resistance. The effects of GO were compared with those of a resveratrol and schizandrin mixture (RS), which is a mixture of the major components of GO. Resveratrol is rich in grapes and is well known to protect against obesity, type 2 diabetes and cardiovascular diseases [8,9]. Schizandrin is the main active ingredient of omija and has been shown to lower blood glucose level and improve insulin resistance in 90% pancreatectomized diabetic rats fed an HFD [10].

## 2. Results and Discussion

**Figure 1:**
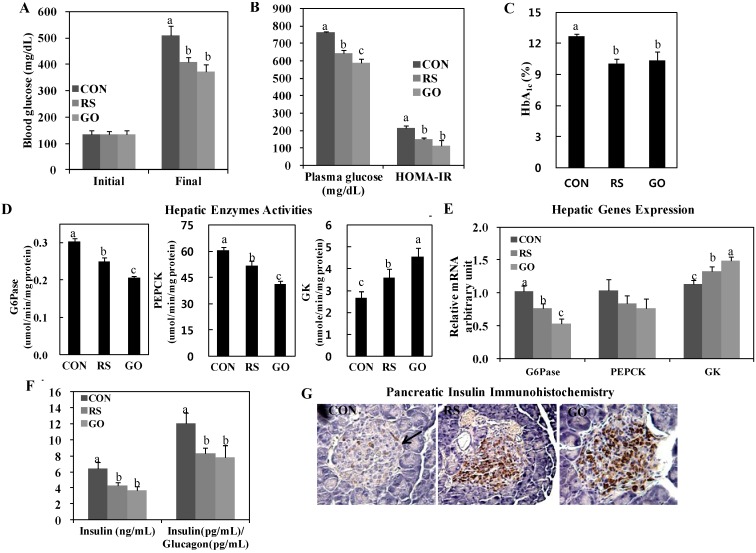
Effects of GO and RS on (**A**) blood glucose; (**B**) plasma glucose and HOMA-IR; (**C**) blood HbA_1c_; (**D**) hepatic enzymes activities; (**E**) hepatic genes expression; (**F**) plasma insulin and insulin/glucagon ratio and (**G**) immunohistochemistry staining for pancreatic insulin in C57BL/KsJ-*db*/*db* mice. **A–F**: Data are the means ± SE (*n* = 10). ^a,b,c^ Means not sharing a common letter are significantly different among the groups at *p* < 0.05; **G**: Representative photomicrographs of the pancreas are shown at 200× magnification (*n* = 10). Arrow, β-cell. CON, normal diet control; RS, normal diet plus resveratrol (0.005%, *w*/*w*) and schizandrin (0.02%, *w*/*w*); GO, normal diet plus grape pomace extract (0.3%, *w*/*w*) combined with omija fruit extract (0.05%, *w*/*w*); HOMA-IR, homeostatic model assessment-insulin resistance = [fasting insulin concentration (mU/L)] × [fasting glucose concentration (mg/dL) × 0.05551]/22.5; G6Pase, glucose-6-phosphatase; PEPCK, phosphoenolpyruvate carboxykinase; GK, glucokinase.

We found that GO significantly lowered the levels of blood and plasma glucose and HbA_1c_ and decreased the homeostasis model assessment of insulin resistance (HOMA-IR) value, similarly to or better than RS supplementation (Figure 1A–C). Interestingly, hepatic glucose-6-phosphatase (G6Pase) and phosphoenolpyruvate carboxykinase (PEPCK) activities were markedly downregulated in GO- and RS-treated groups than in the control, and GO was even more effective than RS in inhibiting hepatic G6Pase and PEPCK activities in the liver (Figure 1D). Moreover, GO and RS treatments significantly increased hepatic glucokinase (GK) activity more than the control (CON) group (Figure 1D). This is consistent with their greater glucose-lowering effects (Figure 1A). GO and RS treatments also significantly increased hepatic GK mRNA expression and decreased hepatic G6Pase mRNA expression than the control (Figure 1E). Hepatic G6Pase and PEPCK activities are elevated in type 2 diabetic animals, such as *db*/*db* mice, when compared to normal animals [11]. However, GK, a key glycolytic enzyme, is downregulated in *db*/*db* mice [12]. Do *et al.* [9] indicated that resveratrol led to alter diabetes-related metabolism in *db*/*db* mice that includes the glycemic control effect of grapes. The GO and RS treatments in this present study affected glucose metabolism directly without altering body weights.

Insulin, which is secreted by pancreatic β-cells, is significantly elevated in insulin resistance and/or decreased β-cell mass to maintain glucose homeostasis [13]. The *db*/*db* mice exhibit hyperinsulinemia to compensate for increased insulin resistance, and hyperglycemia manifests at four to eight weeks of age, following β-cell failure [14]. In this study, GO and RS treatments significantly decreased plasma insulin and the insulin/glucagon ratio in addition to preserving pancreatic β-cells compared to the control (Figure 1F,G). Along with the absolute concentration of plasma insulin, the plasma insulin/glucagon ratio is associated with the regulation of hepatic glucose metabolism, because glucagon stimulates gluconeogenesis and glycogenolysis [15]. We observed that GO preserved islet architecture and increased the pancreatic insulin-stained area in *db*/*db* mice, and the protective effect on pancreatic β-cells was more evident in the GO group than in the RS group (Figure 1G).

**Figure 2:**
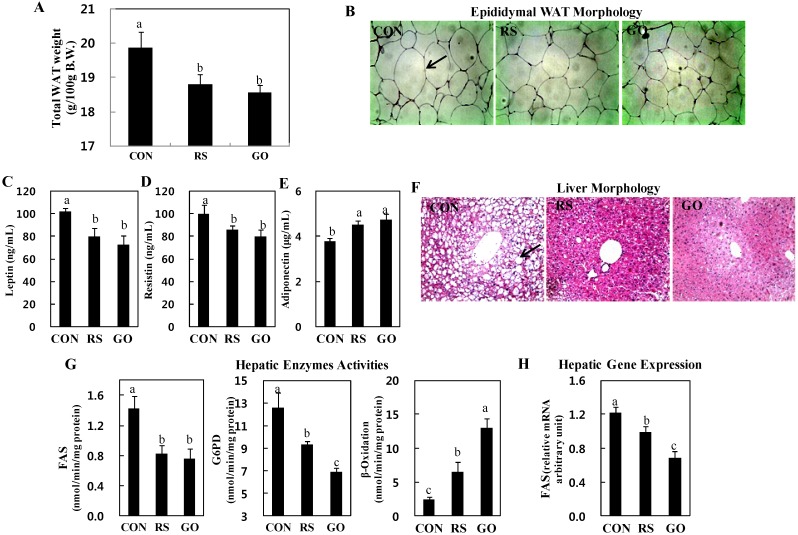
Effects of GO and RS on (**A**) total WAT weight; (**B**) epididymal WAT morphology; (**C**) plasma leptin; (**D**) resistin; (**E**) adiponectin; (**F**) liver morphology; (**G**) hepatic enzymes activities and (**H**) gene expression in C57BL/KsJ-*db*/*db* mice. **A**,**C**–**E**,**G**,**H**: Data are the means ± SE (*n* = 10). ^a,b,c^ Means not sharing a common letter are significantly different among the groups at *p* < 0.05; **B**,**F**: Representative photomicrographs of epididymal WAT and liver are shown at 200× magnification (*n* = 10). Arrows, adipocytes (**B**) and lipid droplets (**F**). CON, normal diet control; RS, normal diet plus resveratrol (0.005%, *w*/*w*) and schizandrin (0.02%, *w*/*w*); GO, normal diet plus grape pomace extract (0.3%, *w*/*w*) combined with omija fruit extract (0.05%, *w*/*w*); WAT, white adipose tissue; FAS, fatty acid synthase; G6PD, glucose-6-phosphate dehydrogenase.

Woods *et al.* [16] have shown that plasma insulin level positively correlates with adiposity, especially visceral adiposity. Adipokines, which are secreted from adipose tissue and include leptin, resistin and adiponectin, have also been proposed as a link among obesity, insulin resistance and diabetes [17]. Leptin positively correlates with body mass index, downregulates β-cell expression of interleukin (IL)-1 receptor antagonist and increases the release of IL-1β in human islets, leading to impaired β-cell function and β-cell death [18]. Resistin induces insulin resistance in islet β-cells, impairs glucose-induced insulin secretion and positively correlates with white adipose tissue (WAT) mass [18]. Adiponectin stimulates glucose utilization and fatty acid oxidation, thereby directly regulating glucose metabolism and insulin sensitivity. In this study, consistent with the plasma insulin levels, the total WAT weights were significantly decreased in GO- and RS-treated groups than in the control (Figure 1F and Figure 2A). The GO- and RS-supplemented mice also had smaller epididymal adipocytes than mice in the control group (Figure 2B). In addition, GO and RS significantly decreased the plasma leptin and resistin levels, but increased plasma adiponectin levels compared to the control (Figure 2C–E).

**Figure 3:**
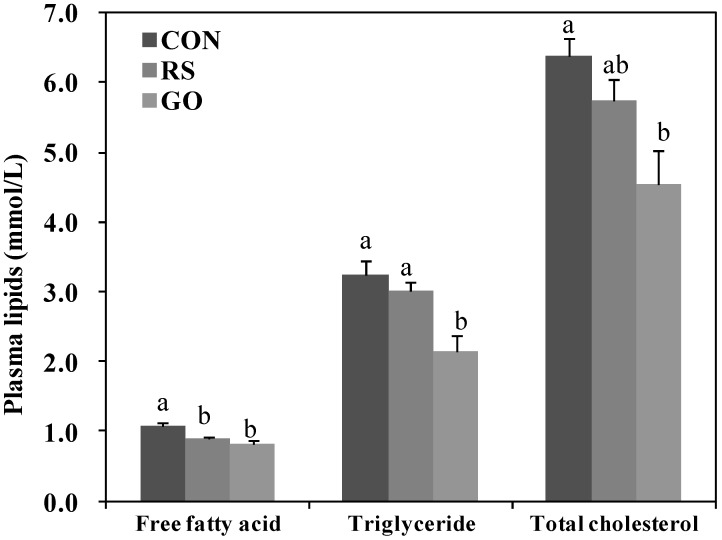
Effects of GO and RS on plasma lipids levels in C57BL/KsJ-*db*/*db* mice.Data are the means ± SE (*n* = 10). ^a,b^ Means not sharing a common letter are significantly different among the groups at *p* < 0.05. CON, normal diet control; RS, normal diet plus resveratrol (0.005%, *w*/*w*) and schizandrin (0.02%, *w*/*w*); GO, normal diet plus grape pomace extract (0.3%, *w*/*w*) combined with omija fruit extract (0.05%, *w*/*w*).

Our previous study showed that grape skin extract ameliorated hepatic steatosis through a decrease in hepatic lipogenic enzyme activity concomitant with β-oxidation activation in HFD-induced obese mice, and schizandrin decreased hepatic cholesterol and triglyceride levels in hypercholesterolemic mice [19,20]. In this study, the GO and RS groups decreased the number and size of lipid droplets in the liver of *db*/*db* mice than the control (Figure 2F). Furthermore, supplementation with GO, as well as RS not only markedly decreased the activities of enzymes for *de novo* fatty acid synthesis, fatty acid synthase (FAS) and glucose-6-phosphate dehydrogenase (G6PD), but also increased fatty acid oxidation in the liver compared to the control (Figure 2G). FAS catalyzes the synthesis of saturated long-chain fatty acid from acetyl-CoA, malonyl-CoA and NADPH, and G6PD is involved in supplying NADPH for fatty acid biosynthesis. Thus, the reduction of hepatic FAS and G6PD activities can limit the availability of long-chain fatty acids that are required for hepatic triglyceride synthesis. Hepatic fatty acid oxidation also diminishes hepatic triglyceride synthesis. On the other hand, circulating lipids can be delivered to the liver and are related to the activities of hepatic lipid-regulating enzymes [21]. The RS and GO treatments lowered plasma free fatty acid (FFA) levels more than the untreated control, and GO also lowered plasma triglyceride and total-cholesterol levels compared to the control (Figure 3).

It is plausible that synergistic effects may exist between total flavonoids and polyphenols of extracts of grape pomace and omija in protective effects on hyperglycemia, adiposity and hepatic steatosis in type 2 diabetic mice. Other unknown active components present in extracts of grape pomace and omija could also play important roles in their biological effects.

## 3. Experimental Section

### 3.1. Materials

Grapes (*Vitis vinifera* L., MBA (Muscat bailey A) species) and omija (*Schisandra chinensis* Baillon) were purchased from Gyeongsangbuk-do, Korea. Resveratrol and schizandrin were purchased from Sigma Chemical Corporation.

### 3.2. Preparation of Extracts

In this study, grape pomace (skin and stem) and omija fruits (*Fructus Schisandrae*) were used. Samples were prepared by adding 2 L of 80% and 50% ethanol to 100 g of dried grape pomace and omija fruit, respectively; extraction was done at 80 °C for 2 h and then cooled. The solution was filtered (Whatman paper No. 2), concentrated with a rotary vacuum evaporator and stored at −70 °C. The final weight of the grape pomace ethanol extract was 19.9 g (recovery rate: 19.9%) and of the omija fruit ethanol extract was 39.7 g (recovery rate: 39.7%), as shown in Supplemental Table S1. Resveratrol and schizandrin are each representative index compounds present in grape pomace and omija, respectively. As shown in Supplemental Table S1, 1 g of grape pomace ethanol extract contains 0.2 mg of resveratrol, 52 mg of total flavonoid and 95 mg of total polyphenol. One gram of omija fruit ethanol extract contains 8 mg of schizandrin, 7 mg of total flavonoid and 32 mg of total polyphenol.

### 3.3. HPLC Analysis of Index Compounds

Index chemical concentrations were simultaneously determined using the HPLC method. The apparatus used was an Agilent 1100/1200 system (Agilent, Waldbronn, Germany) consisting of a binary pump, thermostated autosampler (injection volume = 30 μL and 20 μL, grape pomace ethanol extract and omija fruit ethanol extract, respectively), column oven (set = 35 °C) and a DAD detector (306 and 254 nm, grape pomace ethanol extract and omija fruit ethanol extract, respectively). The column was an Eclipse XDB-C18, 250 mm × 4.6 mm, 5 μm (Agilent). Eluent was acetonitrile and water with a 0.05% (*v*/*v*) TFA gradient. Flow rates were 0.6 and 0.8 mL/min, and running times were 25 min, for grape pomace ethanol extract and omija fruit ethanol extract, respectively. The HPLC chromatograms of schizandrin standard, omija extract, resveratrol standard and grape pomace extract are illustrated in Supplemental Figure S1C, S1D, S2C and S2D.

### 3.4. Animal and Diets

Male C57BL/KsJ-*db/db* mice were purchased from the Jackson Laboratory (Bar Harbor, ME, USA) at 4 weeks of age. The animals were individually housed at a constant temperature (24 °C), a 12 h light/dark cycle and fed a pelletized commercial non-purified diet for 1 week after arrival. The mice were then randomly divided into 3 groups (*n* = 10) and fed the respective experimental diets for 7 weeks: a normal control diet, resveratrol and schizandrin diet (RS, 0.005% resveratrol and 0.02% schizandrin with CON diet, *w*/*w*) and the combined extracts of grape pomace and omija fruit diet (GO, 0.3% grape pomace extract and 0.05% omija fruit extract with CON diet, *w*/*w*). The composition of the diets is presented in Table 1. The mice had *ad libitum* access to food and distilled water during the experimental period. Their food intake and body weight were measured daily and weekly, respectively.

On the 7th week, mice were anaesthetized with diethyl ether and sacrificed after 12 h of fasting. Blood was taken from the inferior vena cava and then centrifuged at 1000 × g for 15 min at 4 °C, and the plasma was separated to analyze the plasma biomarkers. After blood collection, the liver and adipose tissues were promptly removed, rinsed, weighed, frozen in liquid nitrogen and stored at −70 °C. The pancreas was also removed, rinsed and fixed in 1% hydrogen peroxide. This animal study protocol was approved by the Ethics Committee for animal studies at Kyungpook National University, Republic of Korea.

### 3.5. Plasma and Blood Biomarkers

The levels of plasma insulin, glucagon, leptin, resistin and adiponectin were determined with a multiplex detection kit from Bio-Rad (Hercules, CA, USA). All samples were assayed in duplicate and analyzed with a Luminex 200 Labmap system (Luminex, Austin. TX, USA). Data analyses were done with the Bio-Plex Manager software version 4.1.1 (Bio-Rad, Hercules, CA, USA). The blood glucose concentration was measured with whole blood obtained from the tail veins after withholding food for 12 h using a glucose analyzer, GlucDr supersensor (Allmedicus, Korea). The blood glycosylated hemoglobin (HbA_1c_) concentration was measured with an analyzer (MicormatTM I Hemoglobin A_1c_ Test, Bio-Rad, CA), and the plasma glucose level was analyzed with a commercially available kit (Asan, Seoul, Korea). The HOMA-IR was calculated as previously described: HOMA-IR = [fasting insulin concentration (mU/L)] × [fasting glucose concentration (mg/dL) × 0.05551]/22.5 [22]. Plasma lipid concentrations were determined with commercially available kits: total-cholesterol, triglyceride (Asan, Seoul, Korea) and FFA (Wako Chemicals, Richmond, VA, USA).

**Table 1:** Composition of experimental diets (unit: % of diet). CON, normal diet control; RS, normal diet plus resveratrol (0.005%, *w*/*w*) and schizandrin (0.02%, *w*/*w*); GO, normal diet plus grape pomace extract (0.3%, *w*/*w*) combined with omija fruit extract (0.05%, *w*/*w*).

Ingredients	CON	RS	GO
Casein	20	20	20
D,L-Methionine	0.3	0.3	0.3
Sucrose	49.999	49.974	49.649
Cellulose	5	5	5
AIN-mineral ^1^	3.5	3.5	3.5
AIN-vitamin ^2^	1	1	1
Choline bitartrate	0.2	0.2	0.2
Corn Starch	15	15	15
Corn oil	5	5	5
tert-Butylhydroquinone	0.001	0.001	0.001
Resveratrol		0.005	
Schizandrin		0.02	
Grape pomace extract			0.3
Omija fruit extract			0.05
Total	100	100	100

^1^ AIN-76 mineral mixture (grams/kg): calcium phosphate 500, sodium chloride 74, potassium citrate 2220, potassium sulfate 52, magnesium oxide 24, manganous carbonate 3.5, ferric citrate 6, zinc carbonate 1.6, cupric carbonate 0.3, potassium iodate 0.01, sodium celenite 0.01, chromium potassium sulfate 0.55, sucrose 118.03; ^2^ AIN-76 vitamin mixture (grams/kg): thiamin HCl 0.6, riboflavin 0.6, pyridoxine HCl 0.7, niacin 3, calcium pantothenate 1.6, folic acid 0.2, biotin 0.02, vitamin B_12_ 1, vitamin A (500,000 U/gm) 0.8, vitamin D_3_ (400,000 U/gm) 0.25, vitamin E acetate (500 U/gm) 10, menadione sodium bisulfite 0.08, sucrose 981.15.

### 3.6. Hepatic Enzymes Activities

Enzyme sources were prepared according to the method developed by Hulcher and Oleson with slight modification [23]. The GK activity was determined from liver samples homogenized in 9 volumes of a buffer containing 50 mmol/L Tris-HCl, pH 7.4, 100 mmol/L KCl, 10 mmol/L mercaptoethanol and 1 mmol/L EDTA. Homogenates were centrifuged at 100,000× *g* for 1 h; the cytosol was used for the spectrophotometric assay as described by Davidson and Arion, in which the formation of glucose-6-phosphate from glucose at 37 °C was coupled to its oxidation by G6PD and nicotinamide adenine dinucleotide (NAD) [24]. The G6Pase activity was determined in the microsome with a spectrophotometric assay according to the method by Alegre *et al.* [25]. The reaction mixture contained the following: 100 mmol/L sodium Hepes (pH 6.5), 26.5 mmol/L glucose-6-phosphatate and 1.8 mmol/L EDTA, both previously adjusted to pH 6.5, 2 mmol/L NADP^+^, 0.6 IU/L mutarotase and 6 IU/L glucose dehydrogenase. The PEPCK activity was determined according to the method described by Bentle and Lardy [26]. The reaction mixture contained the following in a 1-mL final volume: 77 mol/L sodium Hepes, 1 mmol/L IDP, 1 mmol/L MnCl_2_, 1 mmol/L dithiothreitol, 0.25 mmol/L NADH, 2 mmol/L phosphoenolpyruvate, 50 mmol/L NaHCO_3_ and 7.2 U of malic dehydrogenase. The amount of protein in the enzyme sources was determined with the Bradford method using bovine serum albumin as the standard [27]. Fatty acid β-oxidation activity was measured spectrophotometrically by monitoring the reduction of NAD to NADH in the presence of palmitoyl-CoA, as described by Lazarow [28]. The results were expressed as nmol/min per mg of protein. FAS activity was determined according to the method described by Carl *et al.* through monitoring the malonyl-CoA-dependent oxidation of NADPH at 340 nm, in which the activity represents the oxidized NADPH nmol/min per mg of protein [29]. The G6PD activity was assayed by spectrophotometric methods according to the procedures described by Pitkänen *et al.*, in which the activity was expressed as the reduced NADPH nmol/min per mg of protein [30].

### 3.7. RNA Extraction and Real-Time Quantitative PCR Analysis

Total RNA was isolated from the liver using TRIzol reagent (Invitrogen Life Technologies, Grand Island, NY) according to the manufacturer’s instructions. DNase digestion was used to remove any DNA contamination, and RNA was re-precipitated in ethanol to ensure no phenol contamination. The RNA purity and integrity were evaluated with the Agilent 2100 Bioanalyzer (Agilent Technologies, Palo Alto, CA, USA). Equal amounts of RNA from each experimental group were pooled to normalize individual differences. Total RNA (1 μg) was reverse transcribed into cDNA using the QuantiTect^®^ reverse transcription kit (Qiagen, Berlin, Germany). Then, mRNA expression was quantified by real-time quantitative PCR, using the QuantiTects SYBR green PCR kit (Qiagen, Germany) on the CFX96TM real-time PCR system (Bio-Rad, UK). The sequences of the primers were as follows: FAS, 5'-CGCTCCTCGCTTGTCGTCTG-3' (forward), 5'-AGCCTTCCATC-TCCTGTCATCATC-3' (reverse); G6Pase, 5'-GGAGGAAGGATGGAGGAAGGAATG-3' (forward), 5'-GGTCAGCAATCACAGACACAAGG-3' (reverse); GAPDH (glyceraldehyde-3-phosphate dehydrogenase), 5'-ACAATGAATACGGCTACAGCAACAG-3' (forward), 5'-GGTGGTCCAGGGTTTCTTACTCC-3' (reverse); GK, 5'-CAGGACAGTGGAGCGTGAAGAC-3' (forward), 5'-TTACAGGGAAGGAGAAGGTGAAGC-3' (reverse); PEPCK, 5'-GGAGGAAGGATGGAGGAAGGAATG-3' (forward), 5'-GGTCAGCAATCACAGACACAAGG-3' (reverse). Cycle thresholds were determined based on the SYBR green emission intensity during the exponential phase. The fold changes were calculated using the 2^−∆∆*C*t^ method; transcripts of GAPDH were also amplified from the samples in order to validate the internal control genes for real-time quantitative PCR detection.

### 3.8. Histopathological Analysis

Liver and epididymal fat were removed and fixed in a buffer solution of 10% formalin. Fixed tissues were processed routinely for paraffin embedding, and 4-μm sections were prepared and dyed with hematoxylin-eosin. Stained areas were viewed using an optical microscope with a magnifying power of ×200. For the immunohistochemistry of pancreatic β-cells, the islet was sectioned, fixed in 1% hydrogen peroxide and washed in 0.01 M citrate buffer (pH 6.0). These sections were treated with blocking reagent (Ultra Tech HRP) to prevent nonspecific binding and incubated with monoclonal antibodies against insulin (Santa Cruz Biotech, Inc., Santa Cruz, CA, USA). Antibody reactivity was detected using HRP-conjugated biotin-streptavidin complexes and developed with diaminobenzidine tetrahydrochloride (DAB) as the substrate. Stained areas were viewed using an optical microscope with a magnifying power of ×200.

### 3.9. Statistical Analysis

The statistical analyses were performed with the statistical package for social science software program (SPSS, Inc., Chicago, IL, USA). Significant differences between the means were determined by one-way ANOVA. Duncan’s multiple-range test was performed if differences were identified between the groups at *p* &lt; 0.05. All data are expressed as the mean with its standard error of the mean.

## 4. Conclusions

Our findings clearly indicate that the supplementation of GO ameliorates blood and plasma glucose levels, HbA_1c_ level, HOMA-IR value, adiposity and hepatic steatosis in type 2 diabetic *db*/*db* mice, at least in part, through the preservation of β-cell insulin expression and hepatic insulin sensitivity. Accordingly, the positive metabolic effects of dietary GO could be partly mediated by decreasing glucose and fatty acid synthesis and/or increasing their utilization in the liver by regulating enzyme activities involved in glycolysis, gluconeogenesis, fatty acid synthesis and oxidation (Supplemental Figure S3). Moreover, beneficial metabolic effects are related to improved adipokine production. Further studies are needed to determine the optimal ratios of grape pomace and omija as a dietary supplement in human subjects with high blood glucose.

## Supplementary Materials

Supplementary Figure can be found at http://www.mdpi.com/1422-0067/15/10/17778/s1.

## Supplementary Files

Supplementary File 1
