# Supplementary material for: The Beneficial Effects of Combined Grape Pomace and Omija Fruit Extracts on Hyperglycemia, Adiposity and Hepatic Steatosis in db/db Mice: A Comparison with Major Index Compounds
Source: Int J Mol Sci. 2014 Sep 30;15(10):17778–89. doi: 10.3390/ijms151017778 (PMC4227189; doi:10.3390/ijms151017778)

Supplementary Information

Results

Table S1. The chemical composition of ethanol extracts of grape pomace and omija fruit.

Ethanol Extract	Yield of Extract (%)	Resveratrol	Schizandrin	Total Flavonoid	Total Polyphenol
		(mg/g of Concentrated Plant Ethanol Extract)			
**Grape pomace**	19.9	0.2		52.0	95.0
**Omija fruit**	39.7		8.0	7.0	32.0

Body Weight, Food Intake, Adiposity, Plasma Adipokines and Plasma Lipids Levels

Body weight was gradually increased in all three groups after six weeks and was slightly decreased in the seventh week in all groups. There were no significant differences in the body weight gain and food efficiency ratio (FER) between the groups, although food intake was significantly higher in the control group compared to the other groups (Supplemental Table S2).

Table S2. The effects of RS and GO on body weight gain, food intake and FER levels in C57BL/KsJ-*db/db* mice.

	CON	RS	GO
Initial body weight (g)	31.18 ± 0.56	31.09 ± 0.65	31.09 ± 1.03
Final body weight (g)	46.64 ± 1.26	44.74 ± 0.84	46.11 ± 0.98
Body weight gain (g)	15.30 ± 1.24	13.65 ± 0.80	15.02 ± 1.36
Food intake (g/day)	7.94 ± 0.36 [a]	6.75 ± 0.15 [b]	6.87 ± 0.26 [b]
FER	0.04 ± 0.004	0.04 ± 0.003	0.05 ± 0.005

Data are the mean ± SE (n = 10). [a,b] Means not sharing a common letter are significantly different among groups at $p < 0.05$. CON, normal diet control; RS, normal diet plus resveratrol (0.005%, w/w) and schizandrin (0.02%, w/w); GO, normal diet plus grape pomace extract (0.3%, w/w) combined with omija fruit extract (0.05%, w/w); FER, food efficiency ratio = body weight gain/food intake.

Among the six types of white adipose tissues (WATs), interscapular, subcutaneous and mesentery WATs weights were significantly lower in the normal diet plus grape pomace extract (0.3%, *w*/*w*) combined with omija fruit extract (0.05%, *w*/*w*) (GO) group compared to the normal diet control (CON) group. The subcutaneous WAT weight was also significantly lower in the normal diet plus resveratrol (0.005%, *w*/*w*) and schizandrin (0.02%, *w*/*w*) (RS) group compared to the CON group. As a result, the total WAT weight was significantly lower in the RS and GO groups compared to the CON group (Figure 2A). Furthermore, the RS group, as well as the GO group had a smaller size of epididymal adipocytes compared to the CON control group (Figure 2B).

Figure S1. HPLC chromatogram of schizandrin in omija fruit extract. (**A**) Standard curve of schizandrin; (**B**) structure of schizandrin; (**C**) standard peak of schizandrin; (**D**) peak of schizandrin in omija fruit extract.

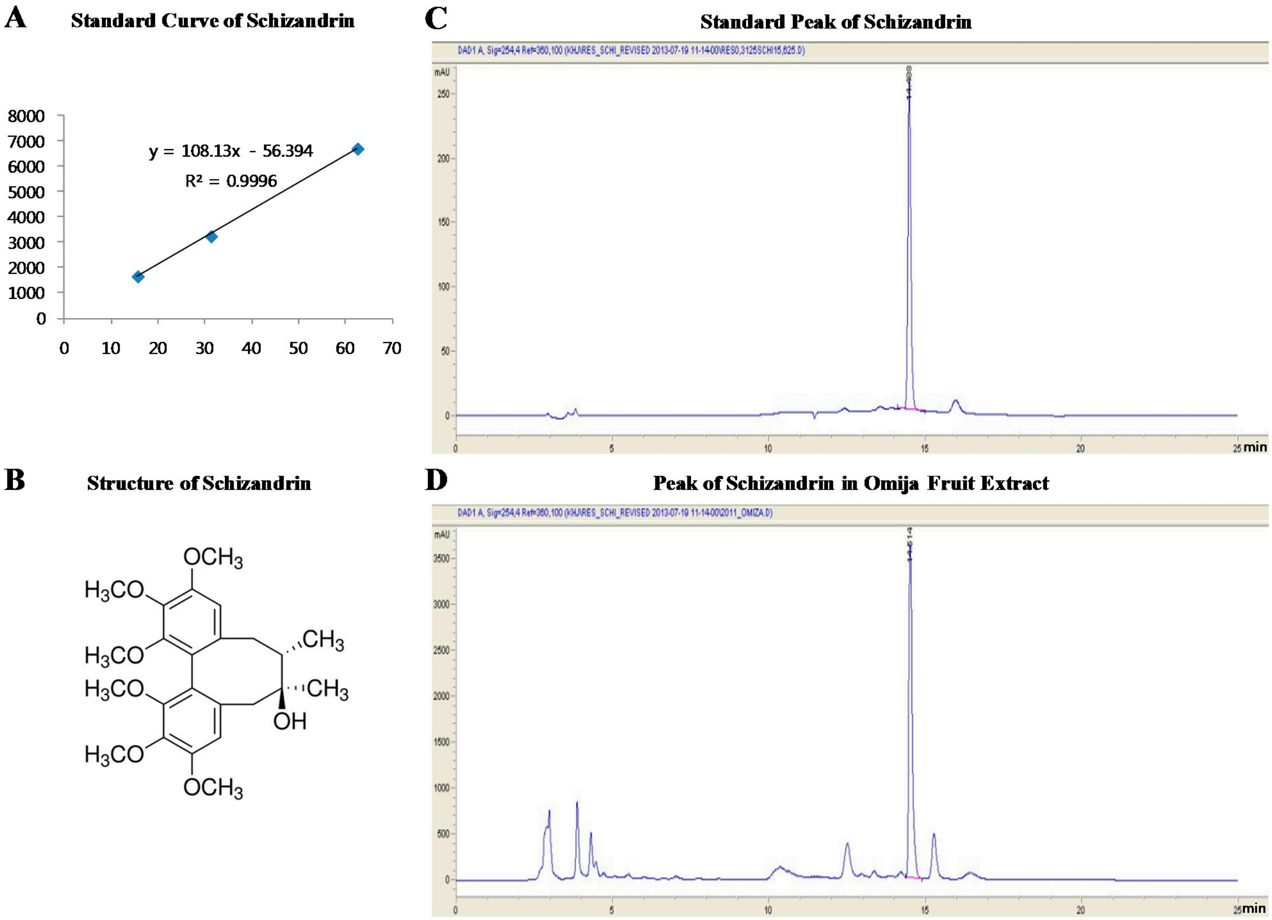

Similar to body fat mass, plasma leptin and resistin levels were significantly lower in the RS and GO groups compared to the CON group, while plasma adiponectin levels were significantly higher in the RS and GO groups compared to the CON group (Figure 2C–E). The plasma free fatty acid level was significantly lower in the RS and GO groups (Figure 3). The GO supplementation also decreased plasma triglyceride and total-cholesterol levels (Figure 3).

Blood Glucose, HbA$_{1c}$, Plasma Glucose, HOMA-IR, Plasma Glucose-Regulating Hormones Levels and Pancreatic Insulin Expression

The initial fasting blood glucose level was similar between the groups (Figure 1A). However, at the end of the experimental period, it was significantly lower in the RS and GO groups than in the CON group (Figure 1A). The levels of blood HbA$_{1c}$ and plasma glucose were significantly lower in the GO group, as well as the RS group compared to the CON group (Figure 1B,C). In particular, the plasma glucose level was significantly lower in the GO group than in the RS group. Homeostasis model assessment of insulin resistance (HOMA-IR) values were also significantly lower in the GO and RS groups compared to the CON group (Figure 1B). Furthermore, the GO group, as well as the RS group significantly lowered the plasma insulin and insulin/glucagon levels compared to the CON group

(Figure 1F). In pancreatic insulin expression by immunohistochemistry, control *db/db* mice exhibited a reduced β-cell population and loss of islet boundary definition together with a decreased insulin-stained area (Figure 1G). The β-cell populations were, however, restored following RS or GO supplementations in *db/db* mice. In particular, mice supplemented with GO showed an increased insulin-stained area with preservation of the pancreatic β-cells compared to the RS-supplemented mice (Figure 1G).

Figure S2. HPLC chromatogram of resveratrol in grape pomace extract. (**A**) Standard curve of resveratrol; (**B**) structure of resveratrol; (**C**) standard peak of resveratrol; (**D**) peak of resveratrol in grape pomace extract.

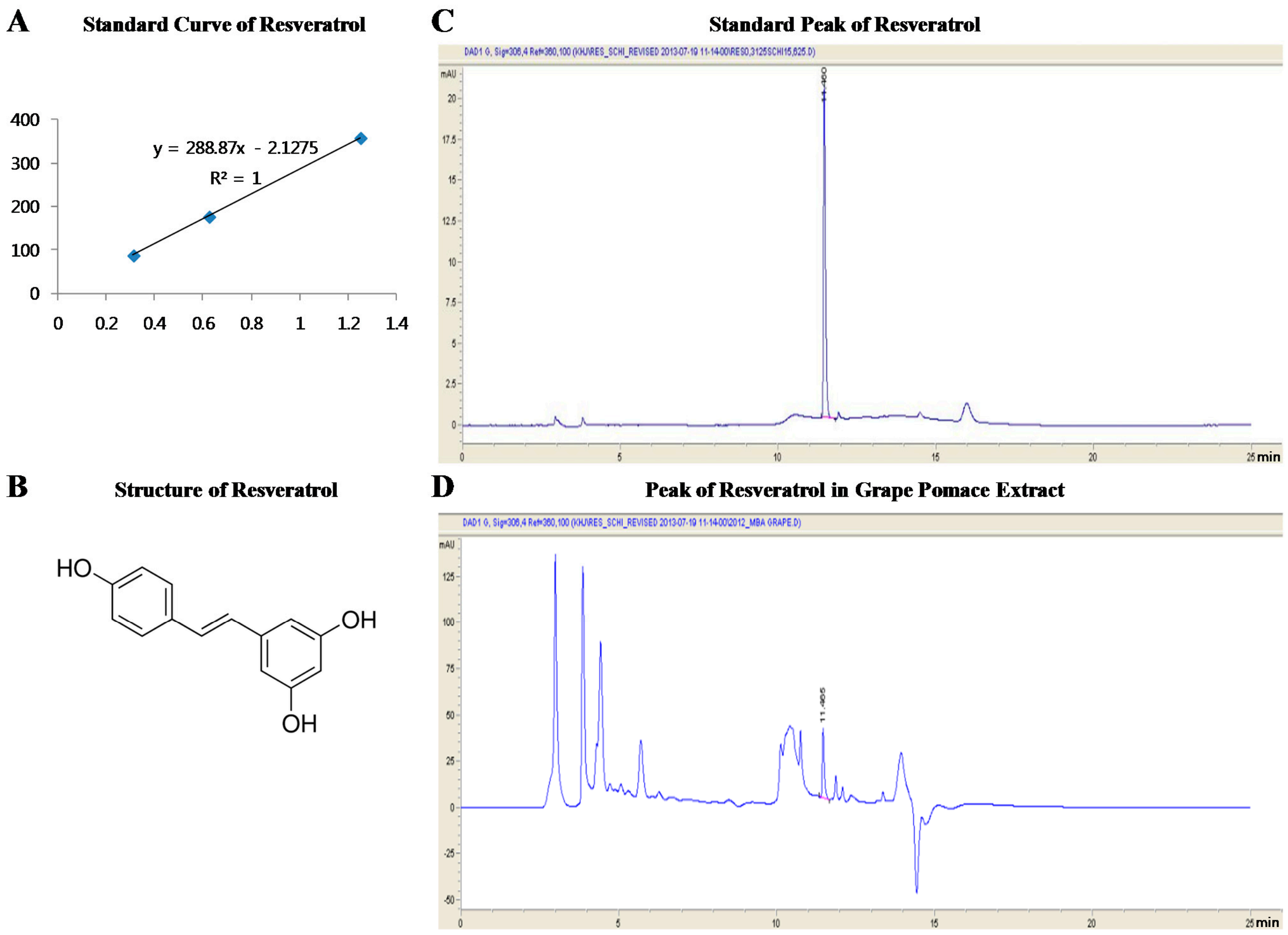

Hepatic Glucose-Regulating Enzymes Activities

To further examine the mechanism by which GO ameliorates hyperglycemia in *db/db* mice, we measured the activities of key gluconeogenic and glycolytic enzymes in liver. The gluconeogenic glucose-6-phosphatase (G6Pase) and phosphoenolpyruvate carboxykinase (PEPCK) activities were significantly lower in the liver of the RS and GO groups compared to the CON group (Figure 1D). In particular, mice supplemented with GO showed a further decrease in hepatic G6Pase and PEPCK activities compared to the RS group. In addition, the RS and GO markedly elevated hepatic glucokinase (GK) activity compared to the CON group (Figure 1D).

Hepatic Lipid Levels and Lipid-Regulating Enzymes Activities

Next, we examined the effect of GO supplementation on hepatic steatosis in *db*/*db* mice. Morphological analysis of the liver revealed that GO reduced the hepatic lipid droplets compared to the CON group, similar to RS (Figure 2F). The *db*/*db* mice fed the supplemented diet with RS or GO also exhibited a significant decrease in hepatic fatty acid synthase (FAS) and glucose-6-phosphate dehydrogenase (G6PD) activities compared to the CON group (Figure 2G). Furthermore, hepatic β-oxidation activities were significantly elevated after supplementation with RS or GO compared to the CON group (Figure 2G).

Expression of Genes in Liver

Supplementation with RS or GO significantly downregulated the mRNA level of G6Pase while upregulating the mRNA level of GK in the liver of *db*/*db* mice fed normal diet (Figure 1E). Hepatic PEPCK expressions were downregulated by 18% and 26% after supplementation with RS and GO compared to the CON group, respectively (Figure 1E). Hepatic lipogenic FAS mRNA levels were significantly downregulated in the RS and GO groups compared to the CON group (Figure 2H).

Figure S3. Summary of the effects of GO on glucose and lipid metabolism in the liver, pancreas and on the WAT of type 2 diabetic mice. GO, normal diet plus grape pomace extract (0.3%, *w*/*w*) combined with omija fruit extract (0.05%, *w*/*w*); WAT, white adipose tissue; FA, fatty acid; FAS, fatty acid synthase; G6PD, glucose-6-phosphate dehydrogenase; PEPCK, phosphoenolpyruvate carboxykinase; G6Pase, glucose-6-phosphatase; GK, glucokinase; HOMA-IR, homeostasis model assessment of insulin resistance.

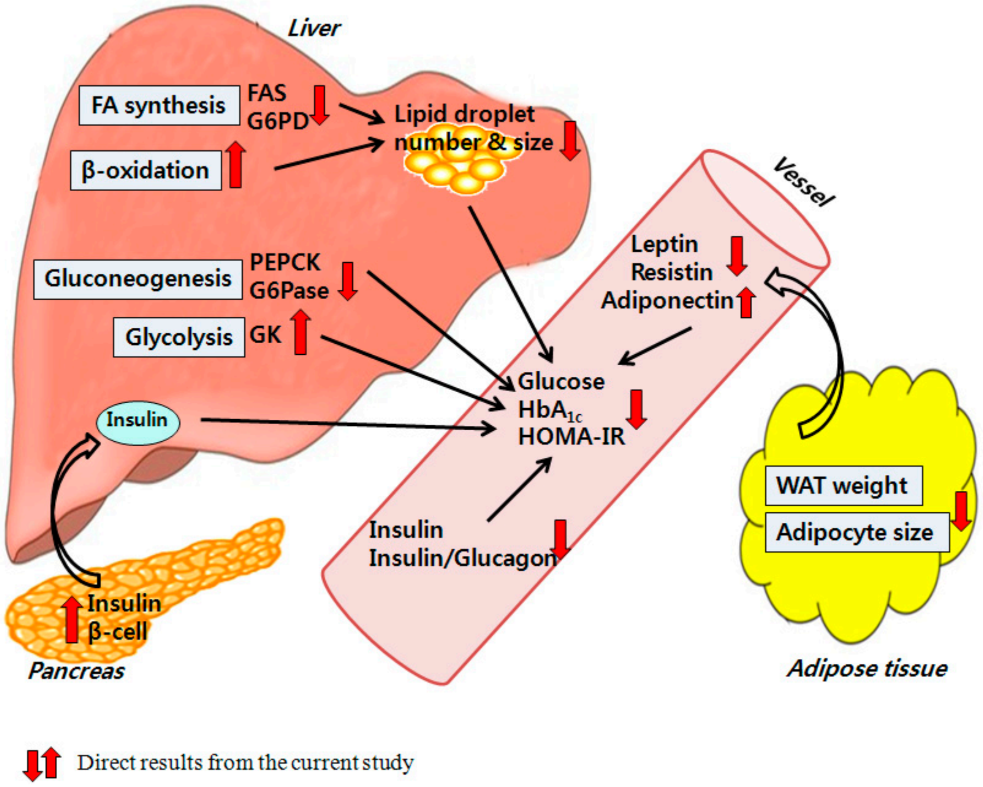

Supplement: Supplementary File 1 [file ijms-15-17778-s001.pdf]
